# Predicting miRNA-Disease Associations Based on Heterogeneous Graph Attention Networks

**DOI:** 10.3389/fgene.2021.727744

**Published:** 2021-08-25

**Authors:** Cunmei Ji, Yutian Wang, Jiancheng Ni, Chunhou Zheng, Yansen Su

**Affiliations:** ^1^School of Cyber Science and Engineering, Qufu Normal University, Qufu, China; ^2^School of Artificial Intelligence, Anhui University, Hefei, China

**Keywords:** disease, miRNA, graph attention networks, miRNA-disease association, DeepWalk

## Abstract

In recent years, more and more evidence has shown that microRNAs (miRNAs) play an important role in the regulation of post-transcriptional gene expression, and are closely related to human diseases. Many studies have also revealed that miRNAs can be served as promising biomarkers for the potential diagnosis and treatment of human diseases. The interactions between miRNA and human disease have rarely been demonstrated, and the underlying mechanism of miRNA is not clear. Therefore, computational approaches has attracted the attention of researchers, which can not only save time and money, but also improve the efficiency and accuracy of biological experiments. In this work, we proposed a Heterogeneous Graph Attention Networks (GAT) based method for miRNA-disease associations prediction, named HGATMDA. We constructed a heterogeneous graph for miRNAs and diseases, introduced weighted DeepWalk and GAT methods to extract features of miRNAs and diseases from the graph. Moreover, a fully-connected neural networks is used to predict correlation scores between miRNA-disease pairs. Experimental results under five-fold cross validation (five-fold CV) showed that HGATMDA achieved better prediction performance than other state-of-the-art methods. In addition, we performed three case studies on breast neoplasms, lung neoplasms and kidney neoplasms. The results showed that for the three diseases mentioned above, 50 out of top 50 candidates were confirmed by the validation datasets. Therefore, HGATMDA is suitable as an effective tool to identity potential diseases-related miRNAs.

## 1. Introduction

MicoRNAs (miRNAs) are a class of endogenous non-coding RNAs with a length of about 21–25 nucleotides, which play an important role in the regulation of post-transcriptional gene expression in organisms (Ambros, 2001, 2004; Bartel, 2004, 2018). Over the past decades, researchers have identified hundreds of miRNAs in humans and shown that many of them interact with most human mRNAs (Friedman et al., 2009). Recent studies have discovered that miRNAs down-regulate gene expression by degrading or silencing targeting mRNAs, thereby affecting many cellular processes, such as growth, development, differentiation, and death (Ambros, 2003; He and Hannon, 2004; Bartel, 2009). Furthermore, many researches have found that human miRNAs are involved in many human diseases (Croce, 2008; Li et al., 2014; Chou et al., 2016; Huang et al., 2019b). Therefore, miRNAs are promising biomarkers for diagnosis and treatment of human diseases.

Based on previous biological experiments, verifying the association between miRNA and disease is time-consuming and expensive. Computational methods can efficiently select the promising disease-associated miRNAs for further experimental verification. Many computational methods have been proposed for predicting the miRNA-disease associations, which can be roughly divided into two categories: one is based on similarity networks, the other is based on machine learning. Jiang et al. (2010) have firstly introduced similarity network to compute the score between disease and miRNA. They constructed functional miRNA network by computing the overlap of the target genes, and applied the hypergeometric distribution to calculate the association score between disease and miRNA. Since then, various network based approaches have been proposed (Chen et al., 2016, 2018c; Pan et al., 2019; Yu et al., 2019). You et al. (2017) have constructed a heterogeneous network, then proposed a novel path based method named PBMDA for inferring the disease-related miRNAs. However, this method only uses sub-graph information for prediction, which can be improved by considering the global information in the heterogeneous graph. Chen et al. (2012) have firstly developed random walk with restart for predicting miRNA-disease associations (RWRMDA). However, the requirement for at least one known related miRNA in inference may limit the application of RWRMDA. Subsequently, many methods based on random walk were proposed to improve the prediction performance.

Machine learning methods have also been introduced in this field. Matrix completion has been widely used in recommendation systems (Koren et al., 2009). Inspired by these, Li et al. (2017) developed a matrix completion based model to predict the disease-related miRNAs (MCMDA). However, it will be failed to predict new disease (or miRNA) that has no connections with the known miRNAs (or diseases). Chen et al. (2018b) proposed a effective method of inductive matrix completion for predicting, which can be applied for new diseases (or miRNAs), named IMCMDA, it achieved better prediction performance than MCMDA. Almost at the same time, Xiao et al. (2018) proposed a novel method called GRNMF, which combined graph Laplacian regularization with non-negative matrix factorization for miRNA-disease associations prediction in the integrated heterogeneous networks. Chen et al. (2018e) introduced low rank matrix decomposition to reduce noises in the datasets, then inferred the associations between miRNAs and diseases in the integrated heterogeneous graph, including disease semantic network, miRNA functional network, the relative GIP kernel networks, and miRNA-disease associations. Chen et al. (2018d) used bipartite recommendation algorithm to generate the association score between disease and miRNA. In addition, there are many other studies based on matrix completion and matrix factorization to predict potential connections between miRNAs and diseases (Shen et al., 2017; Zhong et al., 2018; Yan et al., 2019; Yu et al., 2019). Furthermore, lots of supervised learning algorithms have been introduced in this field. Chen et al. (2019) used decision tree to infer the miRNA-disease associations. SVM-based methods have been used for predicting the potential relations between miRNAs and diseases (Xu et al., 2011; Chen et al., 2018a). Chen et al. (2015) introduced restricted Boltzmann machine for predicting associations and different types between miRNAs and diseases. Chen et al. (2021) integrated matrix completions with neighborhood constraint for prediction.

Recent decades, deep learning based methods have been gradually used in this field. Fu and Peng (2017) proposed a deep ensemble method and adopted stacked autoencoder for obtaining high-level features from integrated similarities, then a three layers neural networks (NN) was used for prediction. Peng et al. (2019) developed a CNN based model named MDA-CNN for prediction. They constructed disease-gene-miRNA networks, and obtained features of diseases and miRNAs by measuring Pearson correlation coefficient with genes. Then auto-encoder was used for feature selection, followed by a convolutional neural networks (CNN) model for further feature extraction between miRNA-disease pairs. Finally, two fully-connected layers was introduced for classification. Xuan et al. (2018) integrated two CNN models to predict correlation score of the miRNA-disease pair. In order to reduce the impact of negative sample missing on the prediction performance, Zhang et al. (2019) constructed two spliced matrices with the known miRNA-disease associations, disease similarity and miRNA similarity, then used variational autoencoder to calculate the unknown values of miRNA-disease pairs. Ji et al. (2021) used two regression models to learn dense vectors from integrated disease and miRNA similarities, applied the reconstruction probability of an autoencoder model for inferring.

Graph neural networks (GNNs) are a class of models that can effectively extract information from its neighborhood in the graph, it has achieved great success in social networks, knowledge graph, biology and so on (Zhou et al., 2020). DimiG considers the tissue-specific expressions as the features of miRNAs and protein coding genes, and then applies graph convolutional network (GCN) in the protein-coding genes and miRNAs networks (Pan and Shen, 2019). Li et al. (2020a) combined GCN and IMC algorithm to identify disease-related miRNAs. However, the input features of diseases and miRNAs were initialized randomly, which reduced the ability of GCN. Long et al. (2021) developed a novel computational model to predict microbe-disease association (GATMDA). It firstly constructed the input features by integrating similarities of diseases and microbes, and a bipartite network of known microbe-disease associations. Then, it used graph attention networks (GAT) for further learning representations of nodes from the graph. Finally, a decoder with inductive matrix completion (IMC) was selected for prediction. Long et al. (2020) also proposed GNN based model to predict human microbe-drug connections. Unlike GATMDA, they adopted RWR method for initial features learning, and then used GNN with random field. Additionally, negative samples were needed for regression training. GAEMDA integrated similarities of diseases and miRNAs as features of nodes, applied a GCN model for further feature extraction, and then used a bilinear decoder for identification (Li et al., 2020b).

In this paper, we proposed a novel computational model (HGATMDA) that combines graph embedding and graph attention networks to infer disease-related miRNAs. Specifically, we first constructed a miRNA-disease heterogeneous graph. Then, we adopted weighted DeepWalk to learn dense representations of miRNAs and diseases from miRNA-miRNA sub-graph and disease-disease sub-graph, respectively. In addition, we utilized graph attention networks (GAT) to further learn the graph structure information from the miRNA-disease sub-graph. Furthermore, we presented a fully-connected neural networks for inferring the potential associations between miRNAs and diseases. Finally, we evaluated the performance of HGATMDA under five-fold cross validation (CV). The results showed that HGATMDA achieved the best area under ROC curve (AUC) of 94.54 ± 0.34%, the area under P-R curve (AUPR) of 94.05 ± 0.18%, Accuracy of 87.02%, Precision of 94.07%, Recall of 90.04%, F1-score of 87.39%. We conducted case studies on three common human diseases such as breast neoplasms, lung neoplasms, and kidney neoplsms, to further evaluate the performance of HGATMDA. For the three diseases, 50 out of top 50 candidates were confirmed by the validation datasets. Experimental results showed that HGATMDA performed better performance than other state-of-the-art methods, and can be used as an efficient and accurate tool to identity the underlying associations between miRNAs and diseases.

## 2. Materials and Methods

### 2.1. Overview

**Figure 1:**
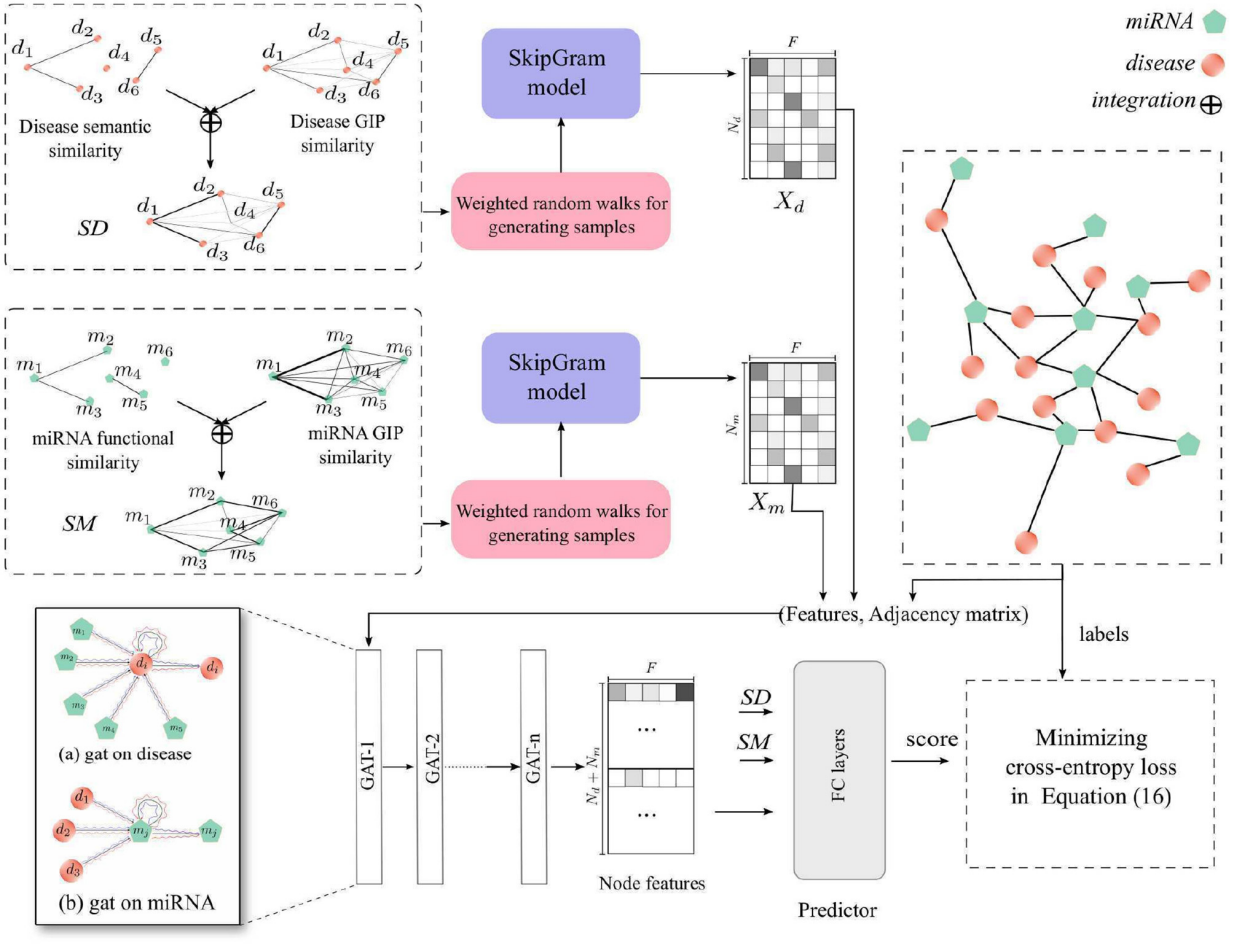
The workflow of HGATMDA for miRNA-disease association prediction.

In this study, we proposed a new computational method, HGATMDA, which combines graph embedding and graph attention network (GAT) methods to identify miRNA-disease associations. As shows in Figure 1, HGATMDA consists of four parts. Firstly, HGATMDA builds a heterogeneous graph including disease-disease sub-graph, miRNA-miRNA sub-graph and miRNA-disease sub-graph. Secondly, HGATMDA adopts a novel weighted DeepWalk to obtain dense representations of diseases and miRNAs from the disease-disease and miRNA-miRNA sub-graphs. Thirdly, it applies GAT to learn node features from the miRNA-disease sub-graph. Lastly, HGATMDA introduces a fully-connected (FC) neural network as an effective classifier for inferring the potential disease-related miRNAs.

### 2.2. Human miRNA Disease Database

HMDD v2.0 (Wang et al., 2010) and HMDD v3.2 (Huang et al., 2019a) databases, the experimental verified human miRNA-disease associations, are used in this paper. We directly downloaded data from http://www.cuilab.cn/hmdd, and constructed a miRNA-disease graph with known associations between miRNAs and diseases. We denote the association matrix as $A^{N_{m}\times N_{d}}$, and *N*_*d*_ is the number of diseases, *N*_*m*_ is the number of miRNAs. The value of *A*_*ij*_ ∈ {0, 1} indicates whether there is a known connections existed between miRNA *m*_*i*_ and disease *d*_*j*_. Here, *N*_*d*_ = 383 and *N*_*m*_ = 495, and 5,430 known interactions between miRNAs and diseases in the HMDD v2.0. We found that the names in HMDD v3.2, including diseases and miRNAs, did not exactly match those in HMDD V2.0. For consistency, we mapped the items in HMDD v3.2 to HMDD v2.0 as the previous work (Wang et al., 2010).

### 2.3. Disease Semantic Similarity

The Medical Subject Headings (MeSH) dataset contains hierarchical relationships between diseases. Tree numbers in the Mesh headings denotes parent-child associations between nodes in the networks. By using the disease name as nodes and tree numbers as edges, we can build a directed acyclic graph (DAG) with the headings for each disease (https://www.nlm.nih.gov/mesh/meshhome.html). We define *DAG*_*d*_ = (*T*_*d*_, *E*_*d*_) as disease *d*, and *T*_*d*_ and *E*_*d*_ are the nodes and edges in the DAG. Then, we can compute the contribution of disease *d*_*i*_ ∈ *T*_*d*_ to disease *d* as follows:

(1) $$\{\begin{array}{ll} D_{d}(d_{i})=1 & \text{if }d=d_{i}\\ D_{d}(d_{i})=max\{\Delta D_{d}(d_{i}^{\prime })|d_{i}^{\prime }\in \text{children of }d_{i}\} & \text{if }d\neq d_{i} \end{array}$$

where Δ is a weight decay parameter and is set as 0.5 in this paper. We define the semantic value of disease *d* by the following formula:

(2) $$\begin{array}{l} DV\left(d\right)=\underset{d^{\prime }\in T_{d}}{\sum }D_{d}\left(d^{\prime }\right) \end{array}$$

Based on the assumption, the more overlap between two DAGs, the more similar the two diseases are. The similarity between disease *d*_*i*_ and *d*_*j*_ can be calculated as follows:

(3) $$\begin{array}{l} SS\left(d_{i},d_{j}\right)=\frac{\sum_{d^{\prime }\in T_{d_{i}}\cap T_{d_{j}}}\left(D_{d_{i}}\left(d^{\prime }\right)+D_{d_{j}}\left(d^{\prime }\right)\right)}{DV\left(d_{i}\right)+DV\left(d_{j}\right)}. \end{array}$$

### 2.4. miRNA Functional Similarity

The basic assumption is that miRNAs with similar functions tend to be connected with similar diseases, and vice versa (Lu et al., 2008; Wang et al., 2010). Wang et al. have proposed miRNA MISIM functional similarity, that is, to calculate the similarity score by the related disease DAG between two miRNAs. Thanks to this excellent work, we can directly download the data from website (http://www.cuilab.cn/files/images/cuilab/misim.zip). We then constructed the miRNA functional network, which is denoted as *FS* with the shape of *N*_*m*_ × *N*_*m*_, where *N*_*m*_ is the number of miRNAs. The element *FS*(*m*_*i*_, *m*_*j*_) represents the similarity score between miRNA *m*_*i*_ and miRNA *m*_*j*_.

### 2.5. Gaussian Interaction Profile Kernel Similarity for Disease and miRNA

In order to overcome the sparsity in disease semantic similarity *SS* and miRNA functional similarity *FS*, we introduced the Gaussian interaction profile (GIP) kernel similarity. Firstly, we used column *A*_·*i*_ to represent a disease *d*_*i*_ and row *A*_*j*·_ to represent miRNA *m*_*j*_. Then, GIP similarities between two diseases or two miRNAs are defined as follows:

(4) $$\begin{array}{l} KD\left(d_{i},d_{j}\right)=\exp\left(-\gamma_{d}||A_{\cdot i}-A_{\cdot j}|{|}^{2}\right) \end{array}$$

(5) $$\begin{array}{l} KM\left(m_{i},m_{j}\right)=\exp\left(-\gamma_{m}||A_{i\cdot }-A_{j\cdot }|{|}^{2}\right) \end{array}$$

here, γ_*d*_ and γ_*d*_ are parameters and can be calculated by following forums:

(6) $$\begin{array}{l} \gamma_{d}=\frac{\gamma_{d}^{\prime }}{\frac{1}{N_{d}}\sum_{i=1}^{N_{d}}||A_{\cdot i}|{|}^{2}} \end{array}$$

(7) $$\begin{array}{l} \gamma_{m}=\frac{\gamma_{m}^{\prime }}{\frac{1}{N_{m}}\sum_{i=1}^{N_{m}}||A_{i\cdot }|{|}^{2}} \end{array}$$

Based on the previous work (van Laarhoven et al., 2011), we set the bandwidth parameters $\gamma_{d}^{\prime }$ and $\gamma_{m}^{\prime }$ to 1 in this paper.

### 2.6. Integrated Similarities for Disease and miRNA

We observed that there was no overlap between DAGs for many diseases, resulting in zero for many elements in disease semantic similarity *SS* and miRNA functional similarity *FS*. While all entries in the GIP similarities *KD* and *KM* are non-zero. Therefore, we integrated GIP similarities with *SS* and *FS* as follows:

(8) $$\begin{array}{l} SD\left(d_{i},d_{j}\right)=\frac{SS\left(d_{i},d_{j}\right)+KD\left(d_{i},d_{j}\right)}{2} \end{array}$$

(9) $$\begin{array}{l} SM\left(m_{i},m_{j}\right)=\frac{FS\left(m_{i},m_{j}\right)+KM\left(m_{i},m_{j}\right)}{2}. \end{array}$$

### 2.7. Weighted DeepWalk for Node Representation

DeepWalk (Perozzi et al., 2014) is an algorithm that can learn the representations of vertices in graphs, inspired by the well-known unsupervised feature learning framework word2vec (Mikolov et al., 2013). Given a graph *G* = (*V, E*), *V* denotes all vertices in the graph, and *E* represents the edge or transaction matrix in the graph. In order to generate the corpus in graphs, a vertex *v*_*i*_ ∈ *V* is uniformly selected as the root, and then a random sampling is used from the neighbors as the next hop. After generating samples, DeepWalk applies SkipGram model to learn the representation of each vertex *v*_*i*_, denotes as $\Phi \left(v_{i}\right)\in {\mathbb{R}}^{d}$. Usually, the dimension size *d* is used as a hyper-parameter.

We applied a variant DeepWalk, called weighted DeepWalk, to learn disease and miRNA representations. The element in *SD* and *SM* can be used as the weight of an edge between two vertices of the disease-disease sub-graph or the miRNA-miRNA sub-graph. Here we used the element in *SD* and *SM* to denote the probability of the current vertex walking to other vertices in the sub-graph. Given a vertex *v*_*i*_, we define a walk sequence *W*_*v*_*i*__ = {*v*_*i*_, ⋯ } to represent the vertices passed by a weighted random walk starting at the vertex *v*_*i*_. We denote the transition possibility of next hop as follows:

(10) $$\begin{array}{l} p\left(v_{j}|v_{i}\right)=p_{ij} \end{array}$$

where *v*_*i*_, *v*_*j*_ ∈ *V* are the vertices in the sub-graph, and *v*_*j*_ ∈ *Ner*_*v*_*i*__ is in the neighbors of vertex *v*_*i*_. *p*_*ij*_ ∈ *SD* or *p*_*ij*_ ∈ *SM* is the weight of an edge from *v*_*i*_ to *v*_*j*_.

After this processing, we obtained two samples {*W*_*d*_1__, *W*_*d*_2__, ⋯ } and {*W*_*m*_1__, *W*_*m*_2__, ⋯ } as the corpus, Each sample is a sentence, and each vertex is a word. For learning the representations of diseases and miRNAs, we used DeepWalk to construct two SkipGram models for learning node representations. There are hundreds of diseases and miRNAs in the respective corpus. Therefore, hierarchical softmax wass used for accelerating the training process. We set the dimension size as *F* in both disease and miRNA representations, and the learned representations of diseases and miRNAs can be denoted as $X_{d}=\left\{d_{1},d_{2},\ldots ,d_{N_{d}}\right\},d_{i}\in {\mathbb{R}}^{F}$, $X_{m}=\left\{m_{1},m_{2},\ldots ,m_{N_{m}}\right\},m_{i}\in {\mathbb{R}}^{F}$. *F* is the dimension size.

### 2.8. Graph Attention Networks for Node Feature Aggregation

In this section, we further constructed a GAT model in the miRNA-disease sub-graph with node features learned from the previous section, to refine dense vectors of miRNAs and diseases. In consequence, these node features contain global heterogeneous graph structure information. We denote the miRNA-disease sub-graph as *G*_*m*−*d*_ = (*V, E*), and the number of nodes is *N*_*d*_ + *N*_*m*_, node *v*_*i*_ ∈ *V* = {*v*_1_, …, *v*_*N*_*d*__, *v*_*N*_*d*_+1_, …, *v*_*N*_*d*_ + *N*_*m*__}, edges (*v*_*i*_, *v*_*j*_) ∈ *E* = {*A*_*ij*_ = 1 ∈ *A*}. The initial node features in the miRNA-disease sub-graph are defined as *X* = [*X*_*d*_, *X*_*m*_], and $x_{i}\in {\mathbb{R}}^{F}$ is the feature of the *i*-th node in the graph. GAT uses multi-head attention mechanism to compute the contributions of neighbors of vertex *v*_*i*_ to itself. We denote the input of *l*-layer of our GAT model as $H^{\left(l\right)}=\left\{h_{1}^{\left(l\right)},h_{2}^{\left(l\right)},\ldots ,h_{N}^{\left(l\right)}\right\},h_{i}^{\left(l\right)}\in {\mathbb{R}}^{F^{\left(l\right)}}$, where *N* = *N*_*d*_+*N*_*m*_ is the number of nodes, and *F*^(*l*)^ denotes the input dimension size of each node. Here, *H*^(0)^ = *X* is the input features of the GAT model. We define the output of *l*-layer as $H^{\left(l+1\right)}=\left\{h_{1}^{\left(l+1\right)},h_{2}^{\left(l+1\right)},\ldots ,h_{N}^{\left(l+1\right)}\right\},h_{i}^{\left(l+1\right)}\in {\mathbb{R}}^{F^{\left(l+1\right)}}$. For each node, we first compute the important score from the neighbor node *j* to node *i*

(11) $$\begin{array}{l} e_{ij}^{\left(l\right)}=a\left({\text{W}}^{\left(l\right)}h_{i}^{\left(l\right)},{\text{W}}^{\left(l\right)}h_{j}^{\left(l\right)}\right) \end{array}$$

where **W**^(*l*)^ is a parameter matrix *W*^(*l*)^ ∈ ℝ^*F*(*l*+1)^ × *F*^(*l*)^, and *a* is a one layer feed-forward neural network. Then, we normalize the neighborhood important scores of node *i* by the softmax function as follows:

(12) $$\begin{array}{l} \alpha_{ij}^{\left(l\right)}={\text{softmax}}_{j}^{\left(l\right)}\left(e_{ij}^{\left(l\right)}\right)=\frac{\exp\left(e_{i,j}^{\left(l\right)}\right)}{\sum_{k\in N_{i}\exp\left(e_{ik}^{\left(l\right)}\right)}} \end{array}$$

where $N_{i}$ is the neighborhood node set of node *i*, including node *i* itself. Finally, we can use these scores to calculate the new features of node *i* by aggregating information from its neighbors:

(13) $$\begin{array}{l} h_{i}^{\left(l+1\right)}=\sigma \left(\underset{j\in N_{i}}{\sum }\alpha_{ij}^{\left(l\right)}{\text{W}}^{\left(l\right)}h_{j}^{\left(l\right)}\right) \end{array}$$

where σ denotes a non-linear activation function, such as LeakyReLU. The power of GAT is benefit from the multi-head attention mechanism, we apply *K* independent attention of node *i* on its neighborhood, and the output of node features is as follows:

(14) $$\begin{array}{l} h_{i}^{\left(l+1\right)}=\sigma \left(\overset{K}{\underset{k=1}{\Lambda }}\underset{j\in N_{i}}{\sum }\alpha_{ij}^{\left(l,k\right)}{\text{W}}^{\left(l,k\right)}h_{j}^{\left(l\right)}\right) \end{array}$$

where Λ denotes concatenation or averaging. In our paper, all GAT layers are used concatenation, except averaging for last layer. A GAT carries out the first-order neighborhood information aggregation, and the graph convolution on multi-layers realizes multi-order neighborhood aggregation. As the number of training iterations increases, the node representations can obtain the structure information of the miRNA-disease sub-graph. Together with the node features obtained from miRNA-miRNA and disease-disease sub-graphs, the final node features of $m_{i}\in {\mathbb{R}}^{F}$ and $d_{j}\in {\mathbb{R}}^{F}$ contain rich structure information of the global heterogeneous graph.

### 2.9. Potential miRNA-Disease Associations Prediction

At last, we designed a scoring function that can calculate the correlation score between a pair of miRNA and disease. We first integrated node features with raw features in *SD* and *SM*. In order to transform the raw features in *SD* and *SM* to the same dimensions as the node features, we introduced projection parameters $W_{d}\in {\mathbb{R}}^{N_{d}\times F}$ and $W_{m}\in {\mathbb{R}}^{N_{m}\times F}$. Then, we can define the correlation score as follows:

(15) $$\begin{array}{l} f\left(d_{i},m_{j}\right)=FC\left(\left[g\left(d_{i},{\left(SD\times W_{d}\right)}_{i\cdot }\right),g\left(m_{j},{\left(SM\times W_{m}\right)}_{j\cdot }\right)\right]\right) \end{array}$$

where *FC* is a fully-connected neural networks, and the details will be discussed in the section 3.3. *g*(·) represents an accumulation function, such as *concat*(·), i.e., a concatenation of node features and raw features, or *sum*(·), i.e., summation of node features and raw features. Our model is trained by minimizing the cross-entropy loss and L2 regularization.

(16) $$\begin{array}{l} \begin{array}{c} L\left(X,Y,\Theta \right)=-\underset{\left(d_{i},m_{j}\right)\in \left\{G^{+}\cup G^{-}\right\}}{\sum }y\log f\left(d_{i},m_{j}\right)\\ \;+\left(1-y\right)\log\left(1-f\left(d_{i},m_{j}\right)\right)+\lambda ||\Theta |{|}^{2} \end{array} \end{array}$$

where Θ denotes parameters of our GAT model, $G^{+}$ is the set of known associations between miRNA and disease, and $G^{-}$ is the same number set of unknown associations between miRNA and disease from negative sampling, which we will discuss the impact in the later section. *X* denotes the vector form of the miRNA-disease pairs in $G^{+}\cup G^{-}$ and *y* ∈ *Y* represents the corresponding label.

## 3. Results

### 3.1. Datasets and Experimental Details

We used benchmark datasets of known miRNA-disease associations, including HMDD, dbDEMC v2.0 (Yang et al., 2017), and miR2Disease (Jiang et al., 2008). Mesh dataset is also used for computing disease semantic similarity. To evaluate the prediction performance of our method, we compared the experimental results with other stat-of-the-art methods. Moreover, we also performed case studies on three common human diseases such as breast neoplasms, lung neoplasms and kidney neoplasms for further evaluation. HMDD v2.0 is used for training, and dbDEMC v2.0, HMDD v3.2, and miR2Disease are used for validation. We carried out experimental analysis using five-fold cross validation. Specially, all known associations in HMDD v2.0 are taken as positive samples. We first selected equal number of negative samples from a uniform distribution of unknown associations between miRNAs and diseases in HMDD v2.0 dataset. Then, we randomly shuffled all the samples and divided into five equal parts, four of which are in turn used for training and the rest for validation. In addition, we applied several metrics for evaluation, such as Accuracy, Precision, Recall, F1-score. Moreover, we plotted the receiver operating characteristic curve (ROC) and precision-recall curve (PR). Furthermore, the area under the ROC curve (AUC), and the area under the P-R curve (AUPR) are used for analysis the prediction performance quantitatively.

The code of our proposed method is implemented based on the machine learning library PyTorch v1.6.0 (Paszke et al., 2019). We trained the weighted DeepWalk models using GenSim library (Rehurek and Sojka, 2010). For GAT, we used PyTorch Geometric deep learning library (Fey and Lenssen, 2019). Our experiments are run on the Ubuntu 16.04 operating system, with two Intel Xeon CPUs (2.30 GHz, 16 cores), and two Tesla V100 GPUs. The Adam optimizer is used for training (Kingma and Ba, 2017), with a learning rate of 0.001 and the weight decay is 0.00005.

### 3.2. Predictive Performance Analysis

**Figure 2:**
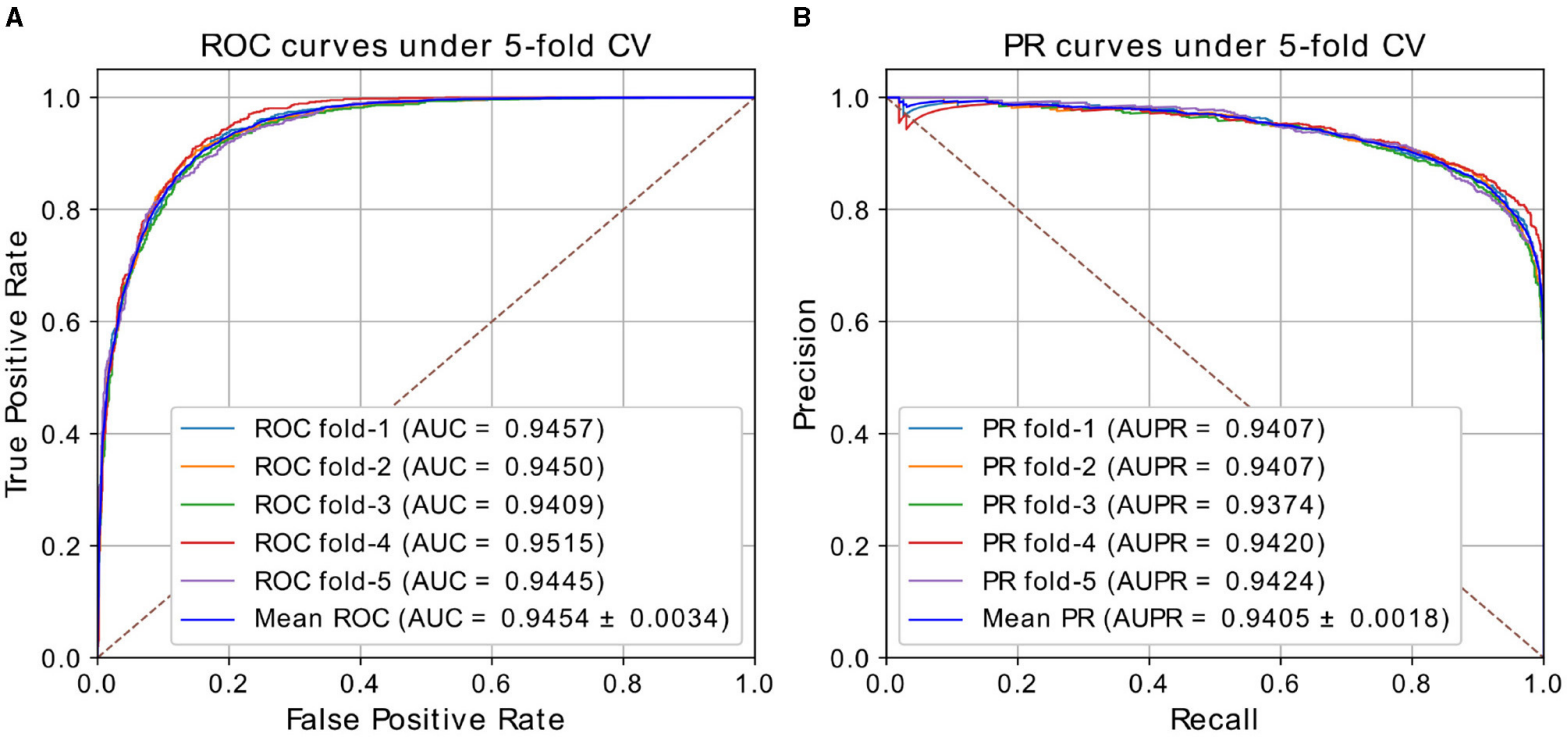
**(A)** ROC curves and **(B)** P-R curves of HGATMDA under five-fold CV based on HMDD v2.0.

In our experiments, we performed ablation study to analyze the effect of architectures and hyper-parameters, then selected the appropriate parameters. Details of the choices will be discussed in the section 3.3. We chose the model with window size of 5, walk length of 20 in weighted Deepwalk, 2 layers in GAT, and 3-layer FC as predictor. The results of our best model under five-fold CV based on HMDD v2.0 dataset are shown in Table 1 and Figure 2. HGATMDA achieves the average prediction Accuracy of 87.02%, Precision of 94.07%, Recall of 90.04%, and F1-score of 87.39%. The threshold used in theses metrics is 0.5. The mean values of AUC and AUPR are 94.54 ± 0.34 and 94.05 ± 0.18%, respectively. In summary, the results showed that HGATMDA can significantly promote the performance of predicting miRNA-disease associations.

**Table 1:** Results of five-fold CV based on HMDD v2.0.

**Test fold**	**Metrics**					
	**Accuracy (%)**	**Precision (%)**	**Recall (%)**	**F1-score (%)**	**AUC (%)**	**AUPR (%)**
1	87.15	94.08	89.87	87.49	94.57	94.07
2	87.57	94.08	89.41	87.79	94.50	94.07
3	86.69	93.75	86.65	86.69	94.09	93.74
4	87.29	94.21	95.30	88.24	95.15	94.20
5	86.37	94.25	88.95	86.71	94.45	94.24
Average	87.02	94.07	90.04	87.39	94.55	94.07

### 3.3. Ablation Experiments

**Figure 3:**
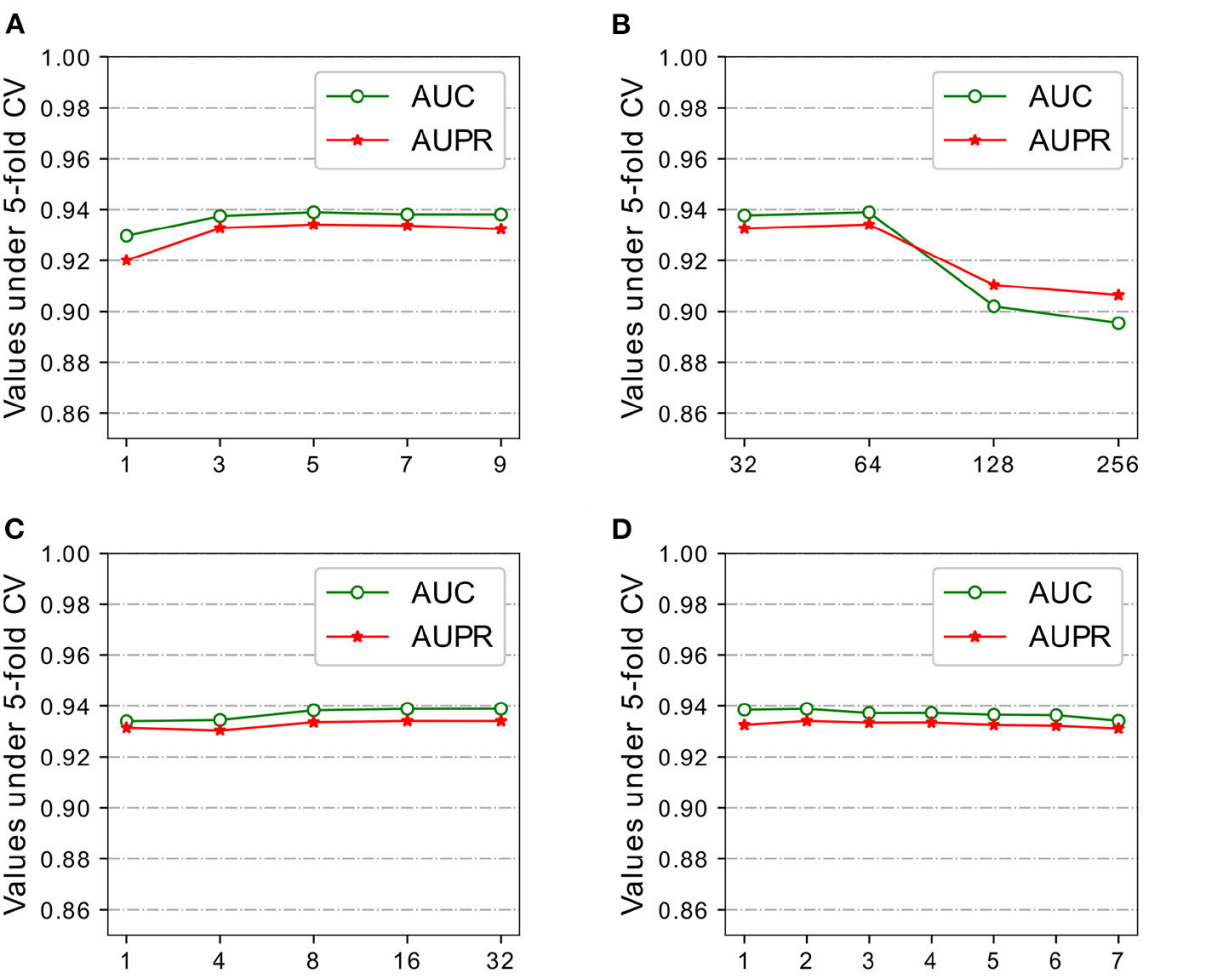
Parameters analysis of DeepWalk and GAT on HMDD v2.0 dataset. **(A)** Window size Weight DeepWalk. **(B)** Feature size Weight DeepWalk. **(C)** # heads of GAT. **(D)** # layers of GAT.

**Figure 4:**
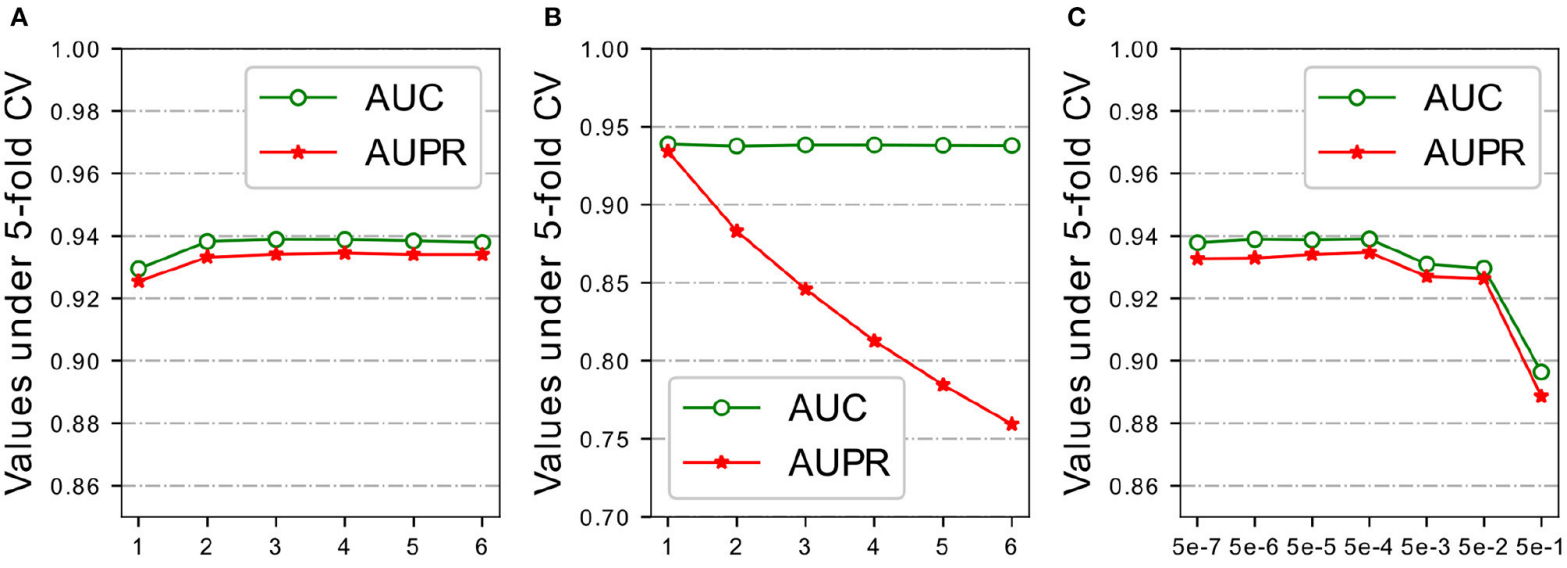
Parameters analysis of FC layers, negative samples and regularization on HMDD v2.0 dataset. **(A)** # FC layers. **(B)** Negative sampling ratio. **(C)** Penalty factor λ.

In order to further demonstrate the prediction performance of our model, we carried out extensive experiments under different architectures and hyper-parameters, analyzed how the design of sub-model and the choice of hyper-parameters have different effect on the final performance of the proposed model. Five-fold CV on HMDD v2.0 dataset was used to evaluate the model sensitivity of architectures and hyper-parameters in the experiments. The following discussion followed the structure in Table 2 and Figures 3, 4. Dot product Predictor denotes the vector dot product of miRNA *m*_*i*_ and disease *d*_*j*_ as the predictor. FC Predictor represents a fully-connected neural networks, as shown in the Equation (15). GAT (untrained) uses randomly weights of neighbors without training, and Raw features denotes integrated similarities of miRNA and disease.

**Table 2:** Ablation experiments of different architectures.

**Test model**	**AUC (%)**	**AUPR (%)**
Weighted DeepWalk + Dot product Predictor	73.48	77.97
Weighted DeepWalk + GAT (untrained) + Dot product Predictor	89.78	88.59
Weighted DeepWalk + GAT + Dot product Predictor	92.17	91.52
Raw features + GAT + Dot product Predictor	72.68	69.27
Weight DeepWalk + Raw features + GAT + Dot product Predictor	93.52	93.09
Weight DeepWalk + Raw features + GAT + FC Predictor	**94.03**	**93.51**

*The variants of the model are listed in the first column. We test each model variant for 10 times under five-fold CV on HMDD v2.0 dataset and report averages. Bold values indicates highlight our results*.

#### 3.3.1. Feature Embedding

Recall that our model adopted weighted DeepWalk to learn representations of miRNAs and diseases from miRNA-miRNA sub-graph and disease-disease sub-graph. We experimented with different feature size of {32,64,128,256} and window size {3,5,7,9} for comparison. Large training windows tend to learn more information from more nodes in the walk, while small training windows do the opposite. HGATMDA obtains the best performance at window size of 5, and the performance slightly decreases with the increase or decrease of the window size (Figure 3A).

Feature size not only affects the representations of miRNAs and diseases in the SkipGram model, but also affects the final prediction performance together with other parameters. We evaluated the effect of feature size using five-fold CV, and mainly focused on values of AUC and AUPR. As shown in Figure 3B, HGATMDA performs best performance when feature size is set to 64 dimensions.

#### 3.3.2. Graph Attention Networks

Representations of miRNAs and diseases are further extract by GAT from miRNA-disease heterogeneous sub-graph. The power of GAT is a multi-head mechanism, we conducted experiments with different number of attention heads. We chose a 2-layer of GAT model, and each layer used same number of heads for comparison. The results are shown in Figure 3C. We found that more heads slightly increased the AUC and AUPR values.

The number of layer is another factor that affects the performance of HGATMDA. We investigated the depth of GAT model used in HGATMDA. Similar to the phenomenon observed in the classification experiments (Kipf and Welling, 2017), we found that the performance of HGATMDA decreased when the number of layer was set to greater than 2. Therefore, we chose 2-layer of GAT with 16 heads as our default.

In the Figure 3D, we can see that using only an untrained GAT architecture greatly improves the prediction performance, achieves 89.78% of AUC and 88.59% of AUPR, more than 10% higher than without GAT.

#### 3.3.3. FC Layers

The predictor used in HGATMDA is a fully-connected neural networks. Given a pair miRNA and disease, the concatenation of representations of the miRNA and disease is fed into the FC layers, to calculate the correlation score between them. We investigated the influence of number of layers in the predictor. The results are shown in Figure 4A, we can see that HGATMDA achieves the better performance when the number of layers is greater than two. Too deep neural network can not improve the predictive performance of HGATMDA. Considering the computation cost, we chose a three-layer neural networks as the predictor in HGATMDA.

#### 3.3.4. Negative Samples

There is lack of prior experimental evidence of non-associations between miRNAs and diseases in the HMDD dataset, we implemented a sampling strategy to randomly generate negative samples from unknown associations in the dataset of HMDD v2.0. As sampling analysis used in the previous study (Long et al., 2020), we experimentally used different sampling ratios to evaluate the effect. Recall that the number of known associations is 5,430, then the number of negative samples is {1.0,2.0,3.0,4.0,5.0,6.0} times the number of positive samples. As shown in Figure 4B, AUC value could not be improved by sampling more negative samples, and the AUPR value is decreased rapidly. More negative samples may result in an imbalance between positive and negative samples, and P-R curve is more sensitive to the difference of category distribution. Therefore, we set the sampling ratio to 1.0 in HGATMDA.

#### 3.3.5. Regularization

Recall that in Equation (16), we introduced the L2 regularization with a penalty factor λ to improve the generalization ability of the model. We conducted experiments with different values of λ to test the effect on prediction performance of the model under five-fold CV. The area under ROC curve and the area under P-R curve are selected as metrics for evaluation. The values we used in this experiment are {0.5, 0.05, 0.005, 0.0005, 0.00005, 0.000005, 0.0000005}. As shown in Figure 4C, We can see that λ is set greater then 0.005, the AUC value declines significantly. Especially, AUC value and AUPR value are both drop below 90% if λ is set as 0.5. When λ is set to 0.0005 or 0.00005, our model achieves better performance. Our model obtains best AUPR at 0.0005, so we finally choose it as our default value.

### 3.4. Comparison With Other Methods

In this section, we compared the prediction performance of our model with other state-of-the-art methods, including PBMDA (You et al., 2017), GRNMF (Xiao et al., 2018), MDHGI (Chen et al., 2018e), BNPMDA (Chen et al., 2018d), MCLPMDA (Yu et al., 2019), NIMCGCN (Li et al., 2020a), and GAEMDA (Li et al., 2020b). We noted that different evaluation metrics and datasets are used in these methods. For fair comparison, AUC values under five-fold CV are selected based on HMDD v2.0 in all these studies for comparison. It is worth noticing that the AUCs reported in these papers are the best values. Therefore, we ran the five-fold CV for 100 times and picked up the best and average AUCs for comparison. The results are shown in Table 3. We can see that our model achieves the best AUC performance among these methods, with the best AUC and average AUC of 94.52 and 93.88%, respectively. In particular, NIMCGCN and GAEMDA are GCN-based methods, and AUCs of our best model are 0.96 and 1.61% higher than these two methods, respectively. This further shows that HGATMDA obtains better performance than other methods.

**Table 3:** The AUCs comparison with other state-of-the-art methods under five-fold CV based on HMDD v2.0.

**Models**	**AUC (%)**
PBMDA (You et al., 2017)	91.72
GRNMF (Xiao et al., 2018)	86.90
MDHGI (Chen et al., 2018e)	87.94
BNPMDA (Chen et al., 2018d)	89.80
MCLPMDA (Yu et al., 2019)	93.25
NIMCGCN (Li et al., 2020a)	92.91
GAEMDA (Li et al., 2020b)	93.56
Our-best	**94.52**
Our-average	**93.88**

*Bold values indicates highlight our results*.

### 3.5. Case Study

We performed case studies on three common human neoplasms, including *Breast Neoplasms, Lung Neoplasms, Kidney Neoplasms*, to further evaluate the prediction performance. In these experiments, HMDD v2.0 dataset is used to train our model, and the validation datasets are HMDD v3.2 (Huang et al., 2019a), dbDEMC v2.0 (Yang et al., 2017), and miR2Disease (Jiang et al., 2008), which are used to verify the candidates. For each specific disease, all known associations in HMDD v2.0 are taken as positive samples, while negative samples are chosen from all unknown miRNA-disease associations except the particular disease related. In the prediction phase, all candidate miRNAs are finally ranked by their correlation scores, which are the last layer of our model with sigmoid activation function, to present how much a miRNA associated with the specific disease. In addition, Top 50 candidates are listed and checked whether they are verified in the validation datasets.

We conducted the first case study for *Breast Neoplasms*. It is reported that Breast cancer is the leading cancer among women worldwide, and can arise for a wide number of reasons. It usually happens when cells in breast tissue grow and divide out of control. As shown in Table 4, 50 out of the top 50 candidates are confirmed in HMDD v3.2, dbDEMC v2.0, or miR2Disease. The second case study was implemented for *Lung Neoplasms*. This is a leading cause of cancer deaths among men and women both in United States and the world (Siegel et al., 2020). We applied our model to predict the most relevant miRNAs with *Lung Neoplasms*. The results are shown in the Table 5, 50 of top 50 selected miRNAs have been verified in the validation datasets. The last case study we selected was *Kidney Neoplasms*. It is another common disease, and the incidence still continues to increase in the United States (Siegel et al., 2020). We note that there are only 7 known associations related with *Kidney Neoplasms* in HMDD v2.0 dataset. We listed top 50 related miRNAs in Table 6, and we can see that 50 out of top 50 candidates are confirmed either in HMDD v3.2, dbDEMC v2.0 or miR2Disease. In particular, 6 of top 10 related miRNAs are verified in at least two datasets. Therefore, our model can serve as a powerful and effective tool to infer the potential related miRNAs for specific diseases.

**Table 4:** Top 50 predicted miRNAs related to Breast Neoplasms based on HMDD v2.0.

**Rank**	**miRNA**	**Evidence**	**Rank**	**miRNA**	**Evidence**
1	hsa-mir-17	*I*; *II*; *III*	26	hsa-let-7c	*I*; *II*
2	hsa-mir-92a	*I*; *II*	27	hsa-mir-125b	*I*; *II*; *III*
3	hsa-mir-1	*I*; *II*	28	hsa-mir-30a	*I*; *II*; *III*
4	hsa-mir-34a	*I*; *II*	29	hsa-mir-143	*I*; *II*; *III*
5	hsa-mir-29a	*I*; *II*	30	hsa-mir-122	*I*; *II*
6	hsa-mir-29c	*I*; *II*; *III*	31	hsa-mir-181a	*I*; *II*; *III*
7	hsa-mir-15a	*I*; *II*	32	hsa-mir-196a	*I*; *II*; *III*
8	hsa-mir-18a	*I*; *II*; *III*	33	hsa-mir-214	*I*; *II*
9	hsa-mir-19b	*I*; *II*	34	hsa-mir-7	*I*; *II*; *III*
10	hsa-mir-155	*I*; *II*; *III*	35	hsa-mir-29b	*I*; *II*; *III*
11	hsa-mir-145	*I*; *II*; *III*	36	hsa-mir-222	*I*; *II*; *III*
12	hsa-mir-16	*I*; *II*	37	hsa-mir-200c	*I*; *II*; *III*
13	hsa-let-7a	*I*; *II*; *III*	38	hsa-mir-9	*I*; *II*
14	hsa-mir-133b	*I*; *II*	39	hsa-mir-100	*I*; *II*
15	hsa-mir-20a	*I*; *II*; *III*	40	hsa-mir-210	*I*; *II*; *III*
16	hsa-mir-133a	*I*; *II*	41	hsa-mir-200b	*I*; *II*; *III*
17	hsa-mir-221	*I*; *II*; *III*	42	hsa-mir-223	*I*; *II*
18	hsa-mir-146a	*I*; *II*; *III*	43	hsa-mir-195	*I*; *II*; *III*
19	hsa-mir-126	*I*; *II*; *III*	44	hsa-let-7b	*I*; *II*
20	hsa-mir-142	*I*; *II*	45	hsa-mir-34c	*I*; *II*
21	hsa-mir-21	*I*; *II*; *III*	46	hsa-let-7f	*I*; *II*; *III*
22	hsa-mir-19a	*I*; *II*	47	hsa-mir-200a	*I*; *II*; *III*
23	hsa-mir-146b	*I*; *II*; *III*	48	hsa-let-7e	*I*; *II*
24	hsa-mir-199a	*I*; *II*	49	hsa-let-7d	*I*; *II*; *III*
25	hsa-mir-150	*I*; *II*	50	hsa-mir-34b	*I*; *II*

*I, II, II denote HMDD v3.2, dbDEMC v2.0, and miR2Disease*.

**Table 5:** Top 50 predicted miRNAs related to Lung Neoplasms based on HMDD v2.0.

**Rank**	**miRNA**	**Evidence**	**Rank**	**miRNA**	**Evidence**
1	hsa-mir-20a	*I*; *II*; *III*	26	hsa-mir-9	*I*; *II*
2	hsa-mir-125b	*I*; *II*; *III*	27	hsa-mir-203	*I*; *II*; *III*
3	hsa-mir-146b	*I*; *II*; *III*	28	hsa-mir-29c	*I*; *II*; *III*
4	hsa-mir-17	*I*; *II*; *III*	29	hsa-mir-155	*I*; *II*; *III*
5	hsa-mir-181a	*I*; *II*; *III*	30	hsa-mir-34c	*I*; *II*; *III*
6	hsa-mir-145	*I*; *II*	31	hsa-mir-142	*I*; *II*; *III*
7	hsa-mir-34a	*I*; *II*; *III*	32	hsa-mir-143	*I*; *II*
8	hsa-mir-29b	*I*; *II*; *III*	33	hsa-mir-30a	*I*; *II*; *III*
9	hsa-mir-146a	*I*; *II*; *III*	34	hsa-mir-221	*I*; *II*; *III*
10	hsa-mir-126	*I*; *II*; *III*	35	hsa-mir-133b	*I*; *II*; *III*
11	hsa-let-7a	*I*; *II*; *III*	36	hsa-mir-31	*I*; *II*
12	hsa-let-7d	*I*; *II*; *III*	37	hsa-mir-182	*I*; *II*; *III*
13	hsa-mir-92a	*I*; *II*; *III*	38	hsa-mir-34b	*I*; *II*; *III*
14	hsa-let-7i	*I*; *II*; *III*	39	hsa-let-7c	*I*; *II*; *III*
15	hsa-mir-19a	*I*; *II*; *III*	40	hsa-mir-122	*I*; *II*; *III*
16	hsa-mir-19b	*I*; *II*	41	hsa-mir-222	*I*; *II*; *III*
17	hsa-let-7b	*I*; *II*	42	hsa-mir-200b	*I*; *II*; *III*
18	hsa-let-7g	*I*; *II*	43	hsa-mir-18a	*I*; *II*
19	hsa-mir-200c	*I*; *II*; *III*	44	hsa-mir-199a	*I*; *II*; *III*
20	hsa-mir-21	*I*; *II*	45	hsa-mir-214	*I*; *II*
21	hsa-mir-210	*I*; *II*	46	hsa-mir-106b	*II*
22	hsa-let-7f	*I*; *II*; *III*	47	hsa-mir-100	*I*; *II*; *III*
23	hsa-mir-200a	*I*; *II*	48	hsa-mir-148a	*I*; *II*; *III*
24	hsa-let-7e	*I*; *II*; *III*	49	hsa-mir-195	*I*; *II*; *III*
25	hsa-mir-16	*I*; *II*	50	hsa-mir-101	*I*; *II*; *III*

*I, II, II denote HMDD v3.2, dbDEMC v2.0, and miR2Disease*.

**Table 6:** Top 50 predicted miRNAs related to Kidney Neoplasms based on HMDD v2.0.

**Rank**	**miRNA**	**Evidence**	**Rank**	**miRNA**	**Evidence**
1	hsa-mir-21	*I*; *II*; *III*	26	hsa-mir-31	*II*
2	hsa-mir-146a	*II*	27	hsa-mir-150	*II*
3	hsa-mir-155	*I*; *II*	28	hsa-let-7a	*II*; *III*
4	hsa-mir-34a	*I*; *II*	29	hsa-mir-143	*II*
5	hsa-mir-125b	*II*	30	hsa-mir-142	*II*
6	hsa-mir-221	*II*	31	hsa-mir-200b	*II*
7	hsa-mir-29a	*II*; *III*	32	hsa-mir-181a	*II*; *III*
8	hsa-mir-20a	*II*; *III*	33	hsa-mir-23a	*II*
9	hsa-mir-15a	*I*; *II*; *III*	34	hsa-mir-15b	*II*
10	hsa-mir-16	*II*	35	hsa-mir-106b	*II*
11	hsa-mir-17	*I*; *II*; *III*	36	hsa-mir-196a	*II*; *III*
12	hsa-mir-29b	*II*; *III*	37	hsa-mir-133b	*II*
13	hsa-mir-92a	*II*	38	hsa-mir-210	*I*; *II*
14	hsa-mir-29c	*II*; *III*	39	hsa-mir-181b	*II*; *III*
15	hsa-mir-145	*II*	40	hsa-mir-146b	*II*
16	hsa-mir-18a	*II*	41	hsa-mir-30a	*II*
17	hsa-mir-223	*II*	42	hsa-mir-24	*II*
18	hsa-mir-122	*II*; *III*	43	hsa-mir-200c	*I*; *II*
19	hsa-mir-19b	*II*; *III*	44	hsa-mir-9	*II*; *III*
20	hsa-mir-126	*I*; *II*; *III*	45	hsa-mir-200a	*I*; *II*
21	hsa-mir-199a	*I*; *II*; *III*	46	hsa-mir-182	*II*
22	hsa-mir-1	*II*	47	hsa-mir-214	*I*; *II*; *III*
23	hsa-mir-133a	*II*	48	hsa-mir-148a	*II*; *III*
24	hsa-mir-19a	*II*	49	hsa-mir-195	*II*
25	hsa-mir-222	*II*	50	hsa-mir-7	*II*

*I, II, II denote HMDD v3.2, dbDEMC v2.0, and miR2Disease*.

## 4. Discussion

Studies have shown that the occurrence and development of many human diseases are related to the abnormal expression of miRNAs. Traditional biological verification of the interactions between miRNAs and diseases are time consuming and expensive. Therefore, computational methods of predicting the disease-related miRNAs can accelerate the identification process, and help us understand the potential mechanism of the interactions between miRNAs and diseases.

In this paper, we presented a computational method for miRNA-disease association prediction based on Heterogeneous Graph Attention Networks, which is superior to other state-of-the-art methods. Specially, we first constructed a heterogeneous graph containing miRNAs and diseases using disease semantic similarity, miRNA functional similarity, the GIP kernel similarities, and known associations between miRNAs and diseases. Then, we proposed a novel method based on weighted DeepWalk that can learn dense feature representation of miRNAs and diseases from the miRNA-miRNA and disease-disease sub-graphs. Furthermore, a GAT based model was implemented for further feature exaction from the miRNA-disease heterogeneous sub-graph, followed by a 3-layer fully-connected neural networks as the predictor. We conducted experiments on five-fold CV, case studies and ablation study. The results demonstrated that our proposed method HGATMDA can serve as an efficient and reliable tool for predicting potential relations between miRNAs and diseases, as well as therapeutics and clinical research.

Our model has achieved high predictive performance mainly for the following reasons. First, GAT was applied to extract node features in the heterogeneous miRNA-disease graph, which is very effective and expressive by leveraging multi-head attention mechanism. Second, initial node features extraction were obtained by weighted DeepWalk, combined with similarity integration, to further improve miRNA and disease characterization. Third, FC-based predictor is suitable and reliable for inferring the potential disease-related miRNAs.

However, there is still room for further improvement. Our constructed heterogeneous graph maybe inaccurate due to the computing equations used in disease and miRNA similarities. In the future, we can directly use the Mesh headings and relationships (https://www.nlm.nih.gov/mesh/meshhome.html) to build disease-disease sub-graph, and extract node features such as Guo et al. (2021). Furthermore, as our future work, we can introduce more biological information, such as miRNA sequence, miRNA-target, protein-protein, and protein-target interactions, to enrich nodes representations, and construct complex heterogeneous graph (Schlichtkrull et al., 2018) with multiple node and edge types, to enhance the prediction performance of our model.

## Data Availability Statement

The original contributions presented in the study are included in the article/supplementary material, further inquiries can be directed to the corresponding author/s.

## Author Contributions

CJ and YS conceived the entire project and wrote the paper. CJ and CZ developed the prediction method. CJ and JN collected data and designed the experiments. CJ and YW analyzed the results. All authors read and approved the final manuscript.

## Conflict of Interest

The authors declare that the research was conducted in the absence of any commercial or financial relationships that could be construed as a potential conflict of interest.

## Publisher's Note

All claims expressed in this article are solely those of the authors and do not necessarily represent those of their affiliated organizations, or those of the publisher, the editors and the reviewers. Any product that may be evaluated in this article, or claim that may be made by its manufacturer, is not guaranteed or endorsed by the publisher.
